# Financial Loss and Depressive Symptoms in University Students During the First Wave of the COVID-19 Pandemic: Comparison Between 23 Countries

**DOI:** 10.3389/ijph.2022.1604468

**Published:** 2022-07-13

**Authors:** Stefano Tancredi, Claudine Burton-Jeangros, René Ruegg, Elena Righi, Anna Kagstrom, Amelie Quesnel Vallee, Arnaud Chiolero, Piet Bracke, Veerle Buffel, Sarah Van De Velde, Stéphane Cullati

**Affiliations:** ^1^ Population Health Laboratory (#PopHealthLab), University of Fribourg, Fribourg, Switzerland; ^2^ Department of Biomedical, Metabolic and Neural Sciences, University of Modena and Reggio Emilia, Modena, Italy; ^3^ Institute of Sociological Research, Geneva School of Social Sciences, University of Geneva, Geneva, Switzerland; ^4^ Department of Social Work, Bern University of Applied Sciences, Bern, Switzerland; ^5^ Third Faculty of Medicine, Charles University, Prague, Czechia; ^6^ National Institute of Mental Health, Prague, Czechia; ^7^ School of Population and Global Health, McGill University, Montreal, QC, Canada; ^8^ Department of Sociology, Faculty of Arts, McGill University, Montreal, QC, Canada; ^9^ Institute of Primary Health Care (BIHAM), University of Bern, Bern, Switzerland; ^10^ Health & Demographic Research Group, Department of Sociology, Ghent University, Ghent, Belgium; ^11^ Centre for Population, Family and Health, Department of Sociology, University of Antwerp, Antwerp, Belgium; ^12^ Department of Readaptation and Geriatrics, Faculty of Medicine, Université de Genève, Geneva, Switzerland

**Keywords:** mental health, university students, depression, financial loss, coronavirus disease 2019

## Abstract

**Objectives:** To assess the association between students’ financial loss and depressive symptoms during the first wave of the coronavirus disease 2019 (COVID-19) pandemic and whether this association varied by countries having different levels of lockdown measures.

**Methods:** This cross-sectional survey, conducted in spring 2020, included 91,871 students from 23 countries. Depressive symptoms were measured using the shortened Center for Epidemiological Studies Depression Scale and information on lockdowns retrieved from the COVID-19 government response tracker. The association between financial loss and depressive symptoms was investigated estimating prevalence ratios (PR) with multilevel Poisson models.

**Results:** Some 13% of students suffered financial loss during the lockdown and 52% had a relatively high depression score, with large between-countries differences. Minimally and maximally adjusted models showed a 35% (PR = 1.35, 95% Confidence Interval (CI) = 1.29–1.42) and 31% (PR = 1.31, 95% CI = 1.26–1.37) higher prevalence of depressive symptoms in students who lost economic resources compared to students with stable economic resources. No substantial differences in the association were found across countries.

**Conclusion:** Depressive symptoms were more frequent among students who suffered financial loss during the pandemic. Policy makers should consider this issue in the implementation of COVID-19 mitigating measures.

## Introduction

The 2019 coronavirus disease pandemic prompted nationwide lockdowns around the globe, introducing several restrictive social regulations including quarantine and physical distancing, which have a profound effect on all aspects of society. The impact was possibly stronger in some strata of the population, notably university students, who had to cope with an altered academic landscape, worrisome career prospects, and possible financial implications as a result of the measures implemented across countries. These challenging circumstances could have increased their mental health suffering [1].

Common mental disorders, such as depressive and anxiety disorders, are distributed along a socioeconomic gradient [2], with the most disadvantaged groups being affected the most due to a lower availability of financial, social or cognitive resources [3] and being more exposed to psychosocial stress [4, 5]. Socioeconomic inequalities also impact the ability to access mental health services, leading to worse outcomes in people with a lower socio-economic status [6]. A history of mental health problems [7, 8], knowing someone infected by Sars-Cov-2 [9], lower social status [10], low perceived social support [11] and less strong family bond [12] have all been found to be associated with an increased prevalence of mental distress among university students during the pandemic. Financial constraints are also a major source of stress: low family income, financial uncertainty, and family financial loss have all shown negative psychological consequences among students throughout the COVID-19 pandemic [13–15].

During the pandemic, countries implemented differing restrictive policies to mitigate the spread and impact of COVID-19 [16]. These measures impacted the lives of university students in several ways. Despite some potential advantages (e.g., the flexibility of remote learning, that allows students to learn at their own pace or to schedule lessons around other daily activities), the necessity to switch to online learning caused by the pandemic presented a number of challenges for university students, such as difficulties to engage during online classes or to develop a sense of belonging due to limited opportunities for socializing [17]. Apart from the effects of social isolation, these measures may also have had an impact on students’ mental health through financial losses. Due to the pandemic, students who worked part-time could have lost their jobs and student’s parents incomes may have significantly decreased. Moreover, online education and work could have increased household expenses of students (bills, internet expenses etc.). Varying degrees of stringency may have had a differentiated impact on students and household financial situations, and little is known about how levels of mitigation policies affected the association between financial loss and mental health in university students. While some countries (e.g., Italy, France) implemented mandatory stay-at-home, business closures and restriction of internal movements, others (e.g., Sweden) relied mainly on minimal restrictive interventions (social distancing guidelines, ban on large gatherings, ban on travels). Some other countries (e.g., Switzerland, Belgium, United Kingdom) implemented in-between policies, with various degrees of restrictions. How these measures relate to students’ financial loss and depressive symptoms has not been systematically assessed in different countries.

Using data from a large cross-sectional online survey conducted in multiple countries, we aimed to assess, (a) the association between students’ loss of economic resources and depressive symptoms during the first wave of the COVID19 pandemic in 23 countries and (b) whether this association varied across countries respective to levels of measures taken to mitigate the spread and impact of COVID-19.

## Methods

### Study Overview

This study is part of the COVID-19 International Student Well-Being Study (C19 ISWS). C19 ISWS is the result of a study design, protocol, and questionnaire developed by a team of the University of Antwerp, Belgium [18]. The study consisted of a cross-sectional online survey conducted in 110 universities in 26 countries. It collected information on students’ well-being during the first wave of the COVID-19 pandemic in the spring of 2020. Data collection was conducted at different times in each country, as detailed in Supplementary Table S1, but in most countries was carried out within a similar time frame. We included bachelor’s, master’s and Ph.D.’s students enrolled at higher education institutions aged 18 years old or above. International and exchange students were included. To assess the level of measures taken in response to COVID-19 for each country, we retrieved data from the University of Oxford coronavirus government response tracker (OxCGRT) [16], a tool that collected information on governments’ policies in response to the pandemic. Specifically, we used data from the OxCGRT’s stringency index, computed from the beginning of the pandemic to the closing date of the survey for each country (Supplemnetary Table S2). A higher index indicated a stricter response.

**FIGURE 1:**
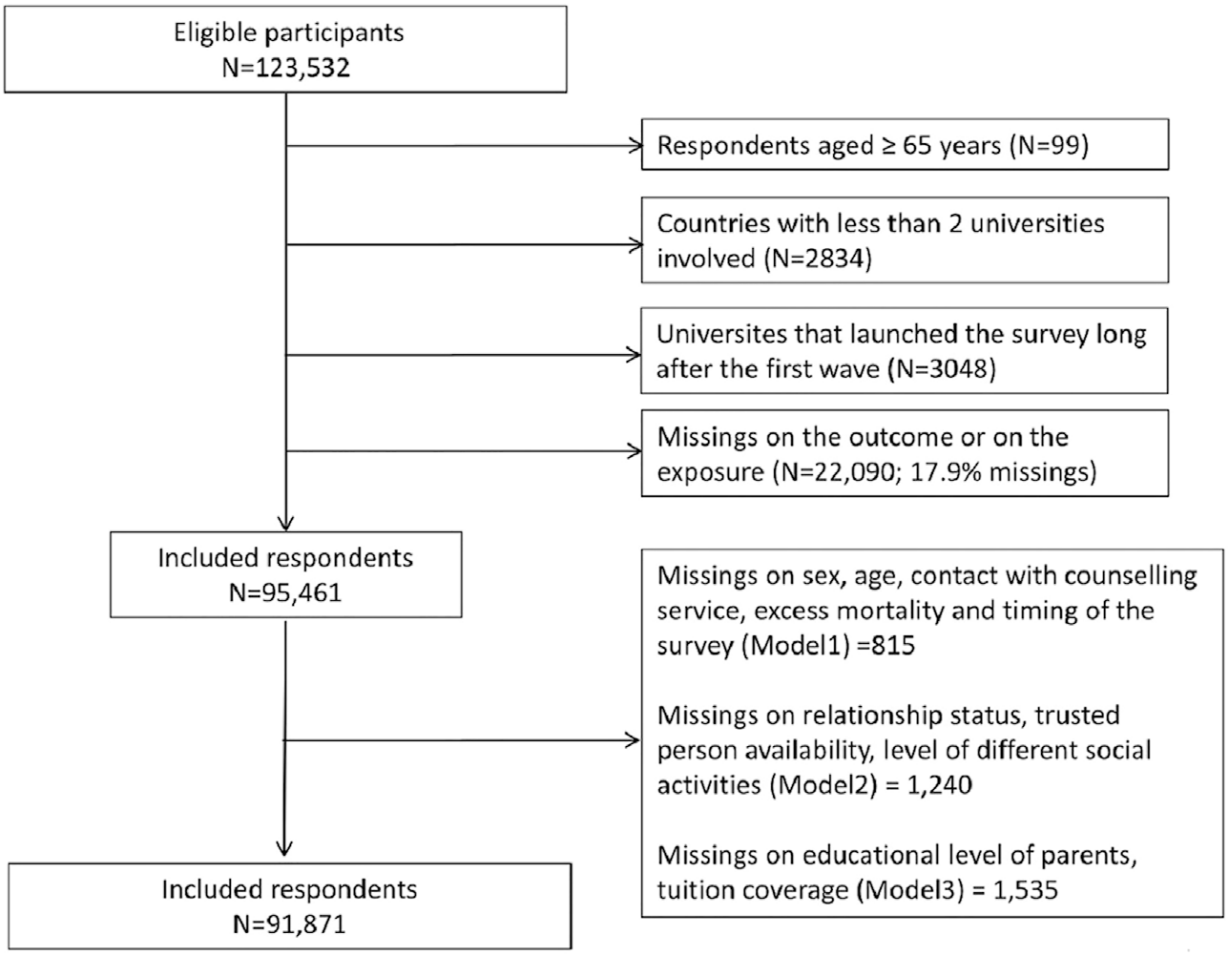
Flow Chart of the respondents’ inclusion, COVID-19 International Student Well-being study, 23 countries worldwide, 2020.

This study followed the Strengthening the Reporting of Observational Studies in Epidemiology (STROBE) guidelines [19]. From the original sample, we excluded participants over 65 years since they could receive a retirement pension, participants from countries with less than two Universities involved in the survey to reduce sampling bias, participants from universities that launched the survey long after the first wave (University of Halle in Germany and University of Amsterdam in Netherlands) and participants with missing data on the outcome variable or on any other analyzed covariates. Figure 1 shows a flow diagram of study participants. Our final sample consisted of 91,871 respondents from 106 universities.

### Measures

#### Outcome: Depressive Symptoms

Self-reported depressive symptoms were assessed using the 8-item version of the Center for Epidemiological Studies Depression Scale (CES-D 8 scale). The original 20-item version was designed to measure depressive symptomatology in the general population [20]. The CES-D 8 scores respondents on a scale of 0–24, and has been found to reliably measure depressive symptomatology in the general population across several European countries [21]. The following questions were used to compute the score: “how much of the time during the past week did you…feel depressed, feel that everything was an effort, sleep restlessly, were happy, feel lonely, enjoy life, feel sad and were unable to get going.” In the main analysis, the CES-D 8 score was dichotomized using a cut-off of 10 as a threshold between low and high depressive levels [22]. We dichotomized the results using 2 additional cut-offs found in the literature [23, 24] to see if the results would change, as detailed in the robustness analysis section.

#### Independent Variable: Loss of Economic Resources

The loss of economic resources was assessed using the questions “To what extent do you agree with the following statement? Before the COVID19 outbreak I had sufficient financial resources to cover my monthly costs” and “During the COVID19 outbreak I had sufficient financial resources to cover my monthly costs.” We dichotomized the answers into agreement vs. disagreement and computed changes between categories. Students who stated that they had sufficient financial resources before the pandemic but not during the pandemic were categorized as students who suffered a financial loss. We categorized the responses using different types of coding schemes, as shown in the robustness analysis section.

#### Covariates

The following covariates were selected based on the scientific literature [25–32] and included in the analysis: Age, sex, contact with a university counselling service since the beginning of the pandemic (Yes vs. No), relationship status (single, in a relationship, it’s complicated), trusted person availability (having anyone with whom discuss intimate and personal matters, Yes vs. No), level of different social activities (frequency of different social activities in the 2 weeks prior the completion of the questionnaire), education levels of parents (high: at least one higher educated parent, low: both parents with less than secondary education, moderate: all other combinations) and tuition coverage (parents paid, self-paid, scholarship, loan, not relevant, other). All covariates were measured using the self-reported questionnaire. To take into account differing states and severity of the pandemic in each country, we also included the following variables: timing of the survey in relation to the peak of the first wave in the country (before, during, after) and excess of mortality at the peak of the first wave (score computed using data from Eurostat or national or regional statistics bureaus [33]).

### Statistical Analyses

#### Main Analyses

In the first phase of data analyses, we examined the distribution of all variables of interest. Frequency tabulation was used to summarize respondents’ information.

For the first aim, we investigated the association between the loss of economic resources and a high depressive symptoms’ score by using prevalence ratios (PR) estimated with multilevel Poisson regression models. Robust variance estimators were used to relax the assumption that the outcome distributions followed a Poisson distribution. We analyzed the data using multilevel models with students (level 1) nested in countries (level 2); results are reported with 95% confidence intervals. We assessed the association between loss of economic resources and a high depressive symptom score through four models. Model 1 was adjusted for sex, age, contact with counselling service, excess of mortality at the peak of the first wave and timing of the survey in relation to the peak of the first wave. Model 2 was additionally adjusted for relationship status/social life (relationship status, trusted person availability and level of different social activities). Based on Model 1, Model 3 was additionally adjusted for socio-economic factors (educational level of parents and tuition coverage). Model 4 was adjusted for all covariates. After fitting the models, we checked for collinearity using the variance inflation factor (VIF).

For the second aim, we tested the role of country level mitigation policies by replicating the same models stratified by country. Statistical analyses were conducted using Stata 16.1 software (Stata Corp, TX, 2019).

#### Robustness Analyses

To compute changes in economic resources, we used 3 different coding schemes. Coding scheme 1 ranged from −4 to 4 (a score of zero meant stable economic resources) and was calculated by scoring from 1 to 5 the possible answers (“strongly disagree,” “disagree,” “neither agree nor disagree,” “agree,” “strongly agree”) to the questions “To what extent do you agree with the following statement? “Before the COVID19 outbreak I had sufficient financial resources to cover my monthly costs” and “During the COVID19 outbreak I had sufficient financial resources to cover my monthly costs” and then subtracting the score of the first question from the score of the second question. To compute coding scheme 2 and 3, we first dichotomized the answers to the same questions into agreement versus disagreement with the “Neither agree nor disagree” response grouped into the agreement category for coding scheme 2 and into the disagreement category for coding scheme 3. Both coding scheme 2 and 3 consisted of 3 categories (increased economic resources, stable economic resources, decreased economic resources).

Moreover, we tested 3 different cut offs for the outcome, used in previous studies. We tested cut offs of 10 and 9 using the CES-D 8 original response format with a score ranging from 0 to 24. We then tested a cut off of 3 dichotomizing the responses of the CES-D 8 scale (“None or almost none of the time” vs. “some of the time” or “most of the time” or “all or almost all of the time”), resulting in a score ranging from 0 to 8. Higher scores indicated a higher frequency of depressive symptoms. We tested our models using all possible combinations of coding schemes and cut-offs. For the main analysis, we used coding scheme 2 and a cut off of 10.

## Results

### Respondents’ Characteristics

The study sample included 91,871 respondents (73% females), with a mean age of 23.3 (SD = 5.54; min: 18; max: 64). Characteristics of the participants stratified by CES-D 8 score are summarized in Table 1. Some 52% of students reported a high depressive symptom score, and 13% of students reported a decrease in their economic resources during the first wave of the COVID-19 pandemic.

**TABLE 1:** Sample characteristics by depression score, COVID-19 International Student Well-being study, 23 countries worldwide, 2020.

	Whole sample (N = 91,871)	Low depressive symptoms’ score (CES-D 8 score<10, N = 43,907)	High depressive symptoms’ score (CES-D 8 score≥10, N = 47,964)
Economic resources, N (%)			
Decreased	11,738 (12.8)	3,645 (31.0)	8,093 (69.0)
Same	78,488 (85.4)	39,658 (50.5)	38,830 (49.5)
Increased	1645 (1.8)	604 (36.8)	1,041 (63.2)
Sex, N (%)			
Male	24,822 (27.0)	13,214 (53.2)	11,608 (46.8)
Female	67,049 (73.0)	30,693 (45.8)	36,356 (54.2)
Age groups, N (%)			
17–18	4,514 (4.9)	2,004 (44.4)	2,510 (55.6)
19–20	23,337 (25.4)	10,227 (43.8)	13,110 (56.2)
21–22	25,572 (27.8)	11,810 (46.2)	13,762 (53.8)
23–24	16,827 (18.3)	8,164 (48.5)	8,663 (51.5)
≥25	21,621 (23.5)	11,702 (54.1)	9,919 (45.9)
Relationship status, N (%)			
Single	43,302 (47.1)	10,145 (44.2)	24,157 (55.8)
In a relationship	43,943 (47.8)	23,306 (53.0)	20,637 (47.0)
It is complicated	4,626 (5.1)	1,456 (31.5)	3,170 (68.5)
Educational level of parents, N (%)			
Low	8,100 (8.8)	3,406 (42.1)	4,694 (57.9)
Moderate	26,393 (29.7)	12,089 (45.8)	14,304 (54.2)
High	57,378 (62.5)	28,412 (49.5)	47,964 (50.5)
Trusted person availability, N (%)			
No	11,612 (12.6)	2,824 (24.3)	8,788 (75.7)
Yes	80,259 (87.3)	41,083 (51.2)	39,176 (48.8)
Tuition coverage (multiple answers allowed), N (%)			
Parents paid	35,758 (38.9)	17,389 (48.6)	18,369 (51.4)
Self-paid	12,987 (14.1)	6,914 (53.2)	6,073 (46.8)
Scholarship	9,289 (10.1)	4,146 (44.6)	5,143 (55.4)
Bank loan or student loan	6,774 (7.4)	2,699 (39.8)	4,075 (60.2)
Not relevant, enrolment is free	23,216 (25.3)	10,902 (47.0)	12,314 (53.0)
Other	3,847 (4.2)	1,857 (48.3)	1,990 (51.7)
Contact with counselling service, N (%)			
Yes	8,250 (9.0)	3,211 (38.9)	5,039 (61.1)
No	83,621 (91.0)	40,696 (48.7)	42,925 (51.3)
Level of different social activities, mean (SD)^a^	4.1 (1.8)	4.2 (1.9)	3.9 (1.8)
Country, N (%)			
Belgium	20,951 (22.8)	9,294 (44.4)	11,657 (55.6)
Québec, Canada	3,991 (4.3)	2,307 (57.8)	1,684 (42.2)
Czech Republic	6,962 (7.6)	3,369 (48.4)	3,593 (51.6)
Denmark	2,271 (2.5)	1,441 (63.5)	830 (36.5)
Finland	1,055 (1.2)	638 (60.5)	417 (39.5)
France	4,171 (4.5)	2,605 (62.5)	1,566 (37.5)
Germany	4,791 (5.2)	2,733 (57.0)	2,058 (43.0)
Greece	584 (0.6)	315 (53.9)	269 (46.1)
Hungary	2,505 (2,7)	1,223 (48.8)	1,282 (51.2)
Iceland	486 (0.5)	343 (70.6)	143 (29.4)
Israel	384 (0.4)	198 (51.6)	186 (48.4)
Italy	9,242 (10.1)	4,576 (49.51)	4,666 (50.5)
Netherlands	10,968 (11.9)	5,248 (47.9)	5,720 (52.2)
Norway	1,934 (2.1)	1,312 (67.8)	622 (32.2)
Portugal	849 (0.9)	411 (48.4)	438 (51.6)
Romania	649 (0.7)	358 (55.2)	291 (44.8)
Russia	2,699 (2.9)	1,171 (43.4)	1,528 (56.6)
South Africa	1,038 (1.1)	352 (33.9)	686 (66.1)
Spain	872 (1.0)	340 (39.0)	532 (61.0)
Switzerland	3,513 (3.8)	2,170 (61.8)	1,343 (38.2)
Turkey	9,739 (10.6)	2,558 (26.3)	7,181 (73.7)
United Kingdom	1,942 (2.1)	785 (40.4)	1,157 (59.6)
Cyprus	275 (0.3)	160 (58.2)	115 (41.8)

a Level of different social activities goes from 0 to 9 and was calculated using the following question “During the last week, did you engage in one of the following activities? Talk on street, recreational class online, game/quiz, video call, talk over phone, chatted, walk, bike ride, drinks/picnic, none.”

SD, standard deviation.

### Loss of Economic Resources and Frequency of Depressive Symptoms

Table 2 shows prevalence ratios for Models 1 to 4. Model 1 showed that students who lost economic resources during the first wave of COVID-19 had a 35% increased prevalence of a high depressive symptoms score compared to students with stable economic resources (PR = 1.35, 95% CI = 1.29–1.42). Adjusting for relationship status/social life and socio-economic factors resulted in a small attenuation of the strength of this association. This attenuation was greater when adjusting for relationship status/social life: differences in PR between Model 1 and Models 2 and 3 were 8.6% and 2.9%, respectively. The maximally adjusted model (Model 4) showed a slight further decrease in the strength of the association for students who lost economic resources who had a 31% increased prevalence of a high depressive symptoms’ score compared to students with equal economic resources (PR = 1.31, 95% CI = 1.26–1.37).

**TABLE 2:** Loss of economic resources and adjusted Prevalence Ratios for depressive symptoms (N = 91,871), COVID-19 International Student Well-being study, 23 countries worldwide, 2020.

	Model 1^a^	Model 2^b^	Model 3^c^	Model 4^d^
	PR (95% CI)	PR (95% CI)	PR (95% CI)	PR (95% CI)
Equal	Ref	Ref	Ref	Ref
Decreased	1.35 (1.29–1.42)	1.32 (1.26–1.38)	1.34 (1.28–1.41)	1.31 (1.26–1.37)

a Model 1 is adjusted for age, sex, contact with counselling service, excess of mortality and timing of the survey.

b Model 2 (Relationship status and social life adjusted) is additionally adjusted for relationship status, trusted person availability, level of different social activities.

c Model 3 (Socio-economic adjusted) = Model 1 + other variables (educational level of parents, tuition coverage).

d Model 4 = Model 1+ all other models’ covariates.

PR, prevalence ratio; 95% CI, 95% Confidence Interval.

### Cross-Country Comparison

**FIGURE 2:**
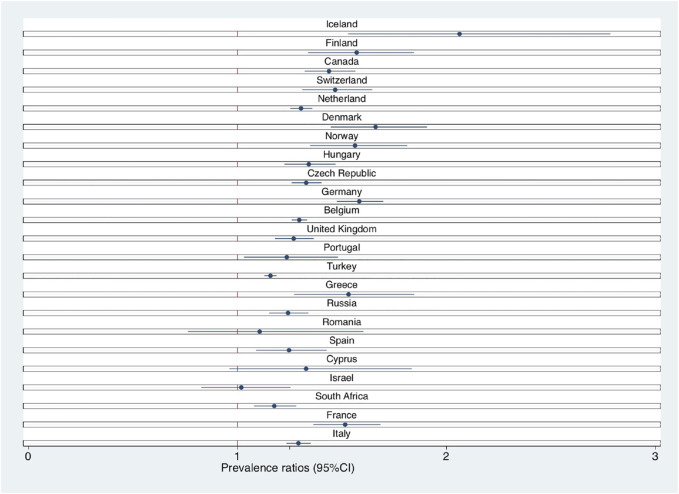
Loss of economic resources and adjusted Prevalence Ratios for depressive symptoms stratified by country, COVID-19 International Student Well-being study, 23 countries worldwide, 2020. Note. Prevalence ratios (PR) were adjusted for age, sex, contact with counselling service, excess of mortality, timing of the survey, relationship status, trusted person availability, level of different social activities, educational level of parents and tuition coverage (see Model 4). Countries are sorted basing on the severity of the lockdown (from the less severe to the most severe) from the beginning of the pandemic to the closing date of the survey for each country.

We observed cross-country differences in the prevalence of students reporting a high depressive symptom score, ranging from 29% (Iceland) to 74% (Turkey), and in the prevalence of students reporting a decrease in their economic resources, as detailed in Supplemental Table S3. Table 3 shows prevalence ratios by country for all Models. Results from Model 1 showed the same trend shown in Table 2 for all countries apart from Israel (PR = 0.99, 95% CI = 0.80–1.23), Romania (PR = 1.10, 95% CI = 0.75–1.61) and Cyprus (PR = 1.36, 95% CI = 0.99–1.86). These results remained consistent for all countries across Models 2 to 4. The strength of the association between the loss of economic resources and the frequency of depressive symptoms had a greater decrease when adjusting for relationship status/social life in all countries apart from Greece, Israel and Spain. Figure 2 shows prevalence ratios of the maximally adjusted model between countries stratified and sorted by intensity of lockdown. No trend depending on lockdown severity was found.

**TABLE 3:** Loss of economic resources and adjusted Prevalence Ratios for depressive symptoms stratified by country, COVID-19 International Student Well-being study, 23 countries worldwide, 2020.

		Model 1^a^	Model 2^b^	Model 3^c^	Model 4^d^
		PR (95%CI)	PR (95%CI)	PR (95%CI)	PR (95%CI)
	Equal	Ref	Ref	Ref	Ref
Belgium	Decreased	1.34 (1.30–1.38)	1.31 (1.28–1.35)	1.32 (1.28–1.36)	1.30 (1.26–1.33)
Québec, Canada	Decreased	1.53 (1.41–1.67)	1.44 (1.32–1.57)	1.53 (1.40–1.66)	1.44 (1.32–1.57)
Czech Republic	Decreased	1.33 (1.26–1.41)	1.33 (1.26–1.40)	1.34 (1.27–1.41)	1.33 (1.26–1.40)
Denmark	Decreased	1.76 (1.53–2.03)	1.68 (1.46–1.93)	1.73 (1.50–1.99)	1.66 (1.45–1.91)
Finland	Decreased	1.63 (1.39–1.91)	1.57 (1.34–1.85)	1.62 (1.38–1.90)	1.57 (1.34–1.84)
France	Decreased	1.57 (1.41–1.74)	1.53 (1.37–1.70)	1.55 (1.40–1.73)	1.52 (1.36–1.68)
Germany	Decreased	1.67 (1.56–1.79)	1.60 (1.49–1.71)	1.65 (1.54–1.77)	1.58 (1.48–1.70)
Greece	Decreased	1.49 (1.23–1.80)	1.53 (1.27–1.83)	1.49 (1.23–1.80)	1.53 (1.27–1.85)
Hungary	Decreased	1.39 (1.27–1.52)	1.34 (1.22–1.47)	1.39 (1.27–1.52)	1.34 (1.22–1.47)
Iceland	Decreased	2.14 (1.59–2.88)	2.07 (1.54–2.79)	2.13 (1.58–2.87)	2.06 (1.53–2.78)
Israel	Decreased	0.99 (0.80–1.23)	1.03 (0.84–1.27)	0.98 (0.79–1.21)	1.02 (0.83–1.25)
Italy	Decreased	1.33 (1.28–1.40)	1.29 (1.24–1.36)	1.33 (1.27–1.39)	1.29 (1.23–1.35)
Netherlands	Decreased	1.36 (1.30–1.41)	1.31 (1.26–1.37)	1.34 (1.29–1.40)	1.30 (1.25–1.36)
Norway	Decreased	1.65 (1.43–1.92)	1.57 (1.36–1.82)	1.64 (1.41–1.90)	1.56 (1.35–1.81)
Portugal	Decreased	1.24 (1.03–1.48)	1.23 (1.03–1.47)	1.24 (1.04–1.49)	1.24 (1.03–1.48)
Romania	Decreased	1.10 (0.75–1.61)	1.09 (0.76–1.56)	1.11 (0.75–1.64)	1.11 (0.76–1.60)
Russia	Decreased	1.26 (1.17–1.36)	1.24 (1.15–1.34)	1.26 (1.17–1.36)	1.24 (1.15–1.34)
South Africa	Decreased	1.23 (1.13–1.34)	1.19 (1.10–1.30)	1.21 (1.11–1.32)	1.18 (1.08–1.28)
Spain	Decreased	1.26 (1.10–1.44)	1.26 (1.10–1.45)	1.24 (1.09–1.41)	1.25 (1.09–1.43)
Switzerland	Decreased	1.57 (1.41–1.76)	1.47 (1.31–1.65)	1.57 (1.40–1.75)	1.47 (1.31–1.64)
Turkey	Decreased	1.18 (1.15–1.21)	1.16 (1.13–1.19)	1.17 (1.14–1.21)	1.16 (1.13–1.19)
United Kingdom	Decreased	1.33 (1.24–1.43)	1.27 (1.18–1.37)	1.32 (1.23–1.42)	1.27 (1.18–1.37)
Cyprus	Decreased	1.36 (0.99–1.86)	1.32 (0.96–1.81)	1.38 (1.00–1.89)	1.33 (0.96–1.83)

a Model 1 is adjusted for age, sex, contact with counselling service, excess of mortality and timing of the survey.

b Model 2 (Relationship status and social life adjusted) is additionally adjusted for relationship status, trusted person availability, level of different social activities.

c Model 3 (Socio-economic adjusted) = Model 1 + other variables (educational level of parents, tuition coverage).

d Model 5 = Model 1+ all other models’ covariates.

PR, prevalence ratio; 95% CI, 95% Confidence Interval.

### Robustness Analyses

Robustness analyses (Supplementary Tables S4–S11) gave similar results as the main analysis. When using coding scheme 1 and a cut-off of 3 differences between different types of adjustments were not found.

## Discussion

This study examined the association between the loss of economic resources and depressive symptoms in university students during the first wave of the COVID-19 pandemic in 23 countries. Some 13% of students experienced a loss in economic resources during the first wave of the COVID-19 pandemic, and we found a relatively high prevalence rate of depressive symptoms. As reported in a review by Brook et al. [34], economic loss was found to be an important post-quarantine stressor associated with mental disorders, with long-lasting consequences. As expected, the results of this study revealed a strong positive association between financial loss and depressive symptoms, confirming the important role of economic constraints as a risk factor related with mental health. Two social mechanisms may explain this association in the context of this study. First, the loss of financial resources may mean the loss (or fear of loss) of “flexible resources” such as power or prestige [3]. These resources could be used to avoid risks or minimize the consequences of a stressful event [35]. In the case of students exposed to the first wave, it is possible that a loss of financial resources meant that they had to draw on their financial reserves, no longer having the availability of a (financial) “cushion” to absorb potential further shocks in the future (a likely scenario at the beginning of the pandemic, when uncertainty was high). Second, the loss of financial resources could lead to an increased level of psychosocial stress, linked to the fear of not being able to make ends meet such as paying bills or buying necessary goods [4, 5]. These two mechanisms are complementary and mutually intertwined. Additionally, we found that adjusting for variables related to students’ social life resulted in a lower strength of the association between financial loss and depressive symptoms compared to adjusting for socioeconomic status (differences in PR between Model 1 and Models 2 and 3 were 8.6% and 2.9%, respectively). As such, support provided by social ties could improve students’ wellbeing [36] and could act as a protective factor in students who experience financial difficulties.

Our results are consistent with other studies in the general population reporting an increased prevalence of depressive symptoms during the pandemic [37]. Although several limitations have to be considered when making comparisons with other studies, our study revealed a high prevalence of depressive symptoms in comparison with past research. Prior to the pandemic, studies have found a 30% mean prevalence of depression for undergraduate students [38] and a 24% prevalence for PhD students [39]. Other studies confirm the positive association between financial loss and depressive symptoms in university students during the pandemic [13–15]. In our study, this association was found in all countries apart from Israel, Romania and Cyprus, with Israel being the only country with a prevalence ratio close to one. However, a previous study conducted in Israel revealed an association between concerns about economic future and risk of depression in the general population [40].

Our second objective was to investigate if the association examined in this study was affected by lockdown severity across countries. Due to lockdowns, university students could have lost their main source of income, such as work-study jobs or part-time jobs, on which students frequently rely to offset some of the costs of higher education. Moreover, students’ parents may have had to interrupt their professional activities, reducing the financial aid they could provide to their children. Although the severity of the lockdowns may be associated with different levels of mental distress, our results did not show a trend between the strength of the association between financial loss and depressive symptoms and the severity of the lockdowns. Possible explanations for this could be that students and their families relied on a financial reserve [41] and/or benefited from government economic support; either of which may have cushioned the financial effect of the lockdowns on mental health during the first phase of the pandemic.

### Limitation and Strengths

When interpreting these results, several limitations should be considered. Firstly, our data represent only the short-term reaction to the first wave of the pandemic, and following lockdowns may have had a different impact on the examined association. In this respect, it should be noted that, although it is likely that the pandemic could have long-term implication on financial resources, there is also evidence of people’s capacity for psychological resilience [42], including a sense of coherence [43], which may reduce the burden of mental health problems associated with the pandemic. Secondly, we used a convenience sample of universities and students, which is not representative of the entire university students’ population within each country. Moreover, we did not have information on students’ history of depressive symptoms. Therefore, we could not assess changes in the frequency of depressive symptoms. Other limitations included selection bias, mainly due to the fact that survey response rates are usually lower for students with worse socioeconomic conditions, and information bias, due to use of the self-reported questionnaire. Furthermore, the results of this study are limited due to its cross-sectional design. In making use of cross-sectional data, causation cannot be inferred.

However, this study capitalized on a large dataset and of reliable information on governments measures taken in response to COVID19, retrieved from the Oxford coronavirus government response tracker. The coordinated effort of the C19-ISWS, which rapidly collected data from different countries across the world during the first phases of the pandemic, allows for cross-country comparisons.

### Conclusion

The findings of this study can help tailor mental health support: economically disadvantaged students should be considered in responses aiming to mitigate the effects of COVID-19 on populations.

When possible, preventing financial hardship experienced by students, or providing targeted economic supports, may help protect their mental health in the context of a pandemic.

## C19 ISWS Consortium Members

Sanna Aaltonen (Department of Social Sciences, University of Eastern Finland, Finland); Thomas Abel (University of Bern, Institute of Social and Preventive Medicine, Switzerland); Petra Arnold (Hungarian Academy of Sciences-Corv, Budapest, Hungary); Iván Balog (Department of Sociology, University of Szeged, Hungary); Péter Balogh (Department of Sociology, University of Szeged, Hungary); Gabriele Berg-Beckhoff (Unit for Health Promotion Research, University of Southern Denmark, Denmark); Jaunathan Bilodeau (Department of Sociology, Faculty of Arts, McGill University, Canada); Marie-Eve Blackburn (Cégep de Jonquière, ECOBES-Research and Transfer, Canada); Marie-Christine Brault (Université du Québec à Chicoutimi, Département des sciences humaines et sociales, Canada); Claudine Burton-Jeangros (University of Geneva, Institute of Sociological Research, Switzerland); Heide Busse (Leibniz Institute for Prevention Research and Epidemiology, Germany); Andreas Chatzittofis (Shacolas Educational Centre for Clinical Medicine, Medical School, University of Cyprus, Cyprus); Fofi Constantinidou (Department of Psychology, University of Cyprus, Cyprus); Anastasia Constantinou (Department of Psychology, University of Cyprus, Cyprus); Adrienne Csizmady (Department of Sociology, University of Szeged, Hungary); Stephane Cullati (University of Fribourg, Population Health Laboratory (#PopHealthLab), Switzerland); Marko J. Elovainio (Department of Psychology and Logopedics, University of Helsinki, Finland); Charles Fleury (Département de sociologie, Université Laval, Canada); María Jesús Rodríguez García (The Urban Governance Lab, Universidad Pablo de Olavide, Spain); Julie Dalgaard Guldager (University of Southern Denmark, Institute of Public Health and University College South, Denmark); Stefanie Helmer (Charité—Universitätsmedizin Berlin, Institute for Health and Nursing Science, Germany); Signe Smith Jervelund (Section of Health Services Research, Department of Public Health, University of Copenhagen, Denmark); Kristiina Kemppainen (Finnish Research Foundation for Studies and Education (OTUS), Finland); Jan Klusacek (Czech Academy of Sciences, Institute of Sociology, Czechia); Michaela Kudrnacova (Czech Academy of Sciences, Institute of Sociology, Czechia); Joel Ladner (School of Medicine, Rouen University Hospital, Rouen University, France); Tina Lauronen (Finnish Research Foundation for Studies and Education (OTUS), Finland); Gyula Lencsés (Department of Sociology, University of Szeged, Hungary); Maria José Guerrero Mayo (The Urban Governance Lab, Universidad Pablo de Olavide, Spain); Rafael Mikolajczyk (Martin Luther University Halle-Wittenberg, Institute for Medical Epidemiology, Biometrics and Informatics, Germany); Cristina Mateos Mora (The Urban Governance Lab, Universidad Pablo de Olavide, Spain); Aliki Mouriki (National Centre for Social Research, Greece); Angel Ramon Zapata Moya (The Urban Governance Lab, Universidad Pablo de Olavide, Spain); Virve Murto (Finnish Research Foundation for Studies and Education (OTUS), Finland); Olga Papaliou (National Centre for Social Research, Greece); Tiina Paunio (Department of Public Health Solutions, National Institute for Health and Welfare, University of Helsinki; SleepWell Research Program, Psychiatry, University of Helsinki and Helsinki University Hospital, Finland); Claudia Pischke (Heinrich Heine University Düsseldorf, Institut fur Medizinische Soziologie, Germany); Charis Psaltis (Department of Psychology, University of Cyprus, Cyprus); René Rüegg (Bern University of Applied Sciences, Switzerland); Kiira Sarasjärvi (University of Helsinki, Tampere University and OTUS, Finland); Nicolas Sauger (Sciences Po, Centre d’études européennes, France); Petr Soukup (Charles University, Institute of Sociological Studies, Czechia); Theoni Stathopoulou (National Centre for Social Research, Greece); Christiane Stock (Charité—Universitätsmedizin Berlin, Institute for Health and Nursing Science, Germany); Christiane Stock (Unit for Health Promotion Research, University of Southern Denmark, Denmark); Marie Pierre Tavolacci (Centre d’Investigation Clinique de Rouen INSERM 1404, School of Medicine, Rouen University Hospital, Rouen University, France); Amelie Quesnel Vallee (Department of Sociology, Faculty of Arts, McGill University, Canada); Anikó Vincze (Department of Sociology, University of Szeged, Hungary); Pia Vuolanto (NEGATE Lab, Faculty of Social Sciences, Tampere University, Finland); Claus Wendt (Universität Siegen, Faculty of Arts, Department of Social Sciences, Germany); Clemente Jesús Navarro Yáñez (The Urban Governance Lab, Universidad Pablo de Olavide, Spain); Hajo Zeeb (Leibniz Institute for Prevention Research and Epidemiology, Germany).
