# Supplementary material for: Financial Loss and Depressive Symptoms in University Students During the First Wave of the COVID-19 Pandemic: Comparison Between 23 Countries
Source: Int J Public Health. 2022 Jul 13;67:1604468. doi: 10.3389/ijph.2022.1604468 (PMC9328628; doi:10.3389/ijph.2022.1604468)
Supplement: Supplementary file 1 [file DataSheet1.docx]

| **Table S1:** recruitment window and sample size per country, COVID-19 International Student Well-being study, 23 countries worldwide, 2020 | | | |
| --- | --- | --- | --- |
| **Country** | **Survey opening and closing date** | **Sample size** | **Number of universities involved** |
| Belgium | 27/04/2020 – 11/05/2020 | 20,951 | 13 |
| Québec, Canada | 07/05/2020 – 27/05/2020 | 3,991 | 4 |
| Cyprus | 03/06/2020 **–** 03/07/2020 | 275 | 2 |
| Czech Republic | 28/04/2020 – 19/05/2020 | 6,962 | 7 |
| Denmark | 11/05/2020 – 05/06/2020 | 2,271 | 2 |
| Finland | 11/05/2020 – 27/05/2020 | 1,055 | 11 |
| France | 11/05/2020 – 24/06/2020 | 4,171 | 2 |
| Germany | 11/05/2020 – 27/05/2020 | 4,791 | 4 |
| Greece | 13/05/2020 – 12/06/2020 | 584 | 7 |
| Hungary | 14/05/2020 – 02/06/2020 | 2,505 | 4 |
| Iceland | 23/06/2020 – 07/07/2020 | 486 | 2 |
| Israel | 30/04/2020 – 20/05/2020 | 384 | 2 |
| Italy | 19/05/2020 – 22/06/2020 | 9242 | 2 |
| Netherlands | 04/05/2020 – 20/05/2020 | 10,968 | 5 |
| Norway | 27/05/2020 – 25/06/2020 | 1,934 | 2 |
| Portugal | 27/04/2020 – 18/05/2020 | 849 | 2 |
| Romania | 28/04/2020 – 27/05/2020 | 649 | 4 |
| Russia | 01/05/2020 – 25/05/2020 | 2,699 | 4 |
| South Africa | 09/06/2020 – 03/07/2020 | 1038 | 4 |
| Spain | 14/05/2020 – 15/06/2020 | 872 | 5 |
| Switzerland | 30/04/2020 **–** 27/05/2020 | 3,513 | 4 |
| Turkey | 11/05/2020 – 31/05/2020 | 9,739 | 11 |
| United Kingdom | 12/05/2020 **–** 01/06/2020 | 1,942 | 2 |
| **All countries** | **27/04/2020 – 07/07/2020** | **91,871** | **106** |

***IJPH- International Journal of Public Health***

**Financial loss and depressive symptoms in university students during the first wave of the COVID-19 pandemic: comparison between 23 countries**

**Table S2**. Oxford COVID-19 Government response tracker’s stringency index, timing of the survey and excess of mortality by country, COVID-19 International Student Well-being study, 23 countries worldwide, 2020

| **Country** | **Oxford stringency index ^a^, mean** | **Timing of the survey in relation to the peak of the first wave** | **Excess of mortality at the peak of the first wave** |
| --- | --- | --- | --- |
| Iceland | 37,16 | After | -0.086154 |
| Finland | 41,08 | After | 0.056631667 |
| Canada | 42,49 | After | 0.41014025 |
| Switzerland | 42,70 | After | -0.021876 |
| Netherlands | 43,38 | After | 0.0240762 |
| Denmark | 44,10 | After | -0.00424575 |
| Norway | 44,94 | After | -0.0346188 |
| Hungary | 45,06 | After | -0.00626325 |
| Czech Republic | 45,90 | During | 0.000967 |
| Germany | 46,08 | After | 0.011046 |
| Belgium | 46,37 | After | 0.184996 |
| United Kingdom | 47,20 | After | 0.2483956 |
| Portugal | 48,27 | After | 0.088520333 |
| Turkey | 49,23 | After | 0.042833667 |
| Greece | 49,56 | After | 0.0215434 |
| Russia | 50,14 | During | 0.4473755 |
| Romania | 52,08 | During | -0.022115 |
| Spain | 53,45 | After | -0.011288167 |
| Cyprus | 54,38 | After | .188657444 |
| Israel | 54,46 | During | 0.1566882 |
| South Africa | 55,38 | Before | 0.13556725 |
| France | 58,38 | After | 0.02645675 |
| Italy | 62,05 | After | -0.052766667 |

^a^: Oxford COVID-19 Government response tracker’s stringency index was computed from the beginning of the pandemic to the closing date of the survey for each country; countries are sorted from the lowest index’s value to the highest value.

| **Table S3.** Prevalence of students with a high depressive symptoms’ score, prevalence of students who suffered financial loss and stringency index by country, COVID-19 International Student Well-being study, 23 countries worldwide, 2020 | | | |
| --- | --- | --- | --- |
| **Country** | **Prevalence of high depressive symptoms’ score (CES-D 8 score≥10)** | **Prevalence of students reporting decreased economic resources** | **Oxford stringency index ^a^, mean** |
| Iceland | 29.4 | 11.5 | 37,16 |
| Finland | 39.5 | 14.1 | 41,08 |
| Canada | 42.2 | 11.3 | 42,49 |
| Switzerland | 38.2 | 7.8 | 42,70 |
| Netherlands | 52.2 | 13.7 | 43,38 |
| Denmark | 36.5 | 6.2 | 44,10 |
| Norway | 32.2 | 11.6 | 44,94 |
| Hungary | 51.2 | 11.0 | 45,06 |
| Czech Republic | 51.6 | 12.1 | 45,90 |
| Germany | 43.0 | 12.5 | 46,08 |
| Belgium | 55.6 | 11.6 | 46,37 |
| United Kingdom | 59.6 | 20.1 | 47,20 |
| Portugal | 51.6 | 10.7 | 48,27 |
| Turkey | 73.7 | 14.8 | 49,23 |
| Greece | 46.1 | 14.4 | 49,56 |
| Russia | 56.6 | 15.4 | 50,14 |
| Romania | 44.8 | 4.2 | 52,08 |
| Spain | 61.0 | 10.1 | 53,45 |
| Cyprus | 41.8 | 16.0 | 54,38 |
| Israel | 48.4 | 35.7 | 54,46 |
| South Africa | 66.1 | 25.1 | 55,38 |
| France | 37.5 | 7.6 | 58,38 |
| Italy | 50.5 | 16.1 | 62,05 |

^a^: Oxford COVID-19 Government response tracker’s stringency index was computed from the beginning of the pandemic to the closing date of the survey for each country

**Table S4**. Loss of economic resources and adjusted Prevalence Ratios for depressive symptoms (N=91,871), coding scheme 3, cut-off:10; COVID-19 International Student Well-being study, 23 countries worldwide, 2020

|  | **Model 1** ^a^ | **Model 2^b^** | **Model 3^c^** | **Model 4** ^d^ |
| --- | --- | --- | --- | --- |
|  | PR (95% CI) | PR (95% CI) | PR (95% CI) | PR (95% CI) |
| Equal | ref | ref | ref | ref |
| Decreased | 1.28 (1.22-1.35) | 1.26(1.21-1.32) | 1.28(1.22-1.34) | 1.26 (1.21-1.32) |

*Abbreviations: PR, Prevalence Ratio; 95% CI, 95% Confidence Interval.

a Model 1 is adjusted for age, sex, contact with counselling service, excess of mortality and timing of the survey

b Model 2 (Relationship status and social life adjusted) is additionally adjusted for relationship status, trusted person availability, level of different social activities

c Model 3 (Socio-economic adjusted) = Model 1 + other variables (educational level of parents, tuition coverage)

d Model 4=Model 1+ all other models’ covariates

**Table S5**. Loss of economic resources and adjusted Prevalence Ratios for depressive symptoms (N=91,871), coding scheme 1, cut-off:10; COVID-19 International Student Well-being study, 23 countries worldwide, 2020

|  | **Model 1** ^a^ | **Model 2^b^** | **Model 3^c^** | **Model 4** ^d^ |
| --- | --- | --- | --- | --- |
|  | PR (95% CI) | PR (95% CI) | PR (95% CI) | PR (95% CI) |
| Coding scheme 1 | 1.11 (1.08-1.13) | 1.10(1.08-1.12) | 1.10(1.08-1.13) | 1.10 (1.08-1.12) |

*Abbreviations: PR, Prevalence Ratio; 95% CI, 95% Confidence Interval.

a Model 1 is adjusted for age, sex, contact with counselling service, excess of mortality and timing of the survey

b Model 2 (Relationship status and social life adjusted) is additionally adjusted for relationship status, trusted person availability, level of different social activities

c Model 3 (Socio-economic adjusted) = Model 1 + other variables (educational level of parents, tuition coverage)

d Model 4=Model 1+ all other models’ covariates

**Table S6**. Loss of economic resources and adjusted Prevalence Ratios for depressive symptoms (N=91,871), coding scheme 1, cut-off:9; COVID-19 International Student Well-being study, 23 countries worldwide, 2020

|  | **Model 1** ^a^ | **Model 2^b^** | **Model 3^c^** | **Model 4** ^d^ |
| --- | --- | --- | --- | --- |
|  | PR (95% CI) | PR (95% CI) | PR (95% CI) | PR (95% CI) |
| Coding scheme 1 | 1.09 (1.07-1.11) | 1.08(1.07-1.10) | 1.09(1.07-1.11) | 1.08 (1.07-1.10) |

*Abbreviations: PR, Prevalence Ratio; 95% CI, 95% Confidence Interval.

a Model 1 is adjusted for age, sex, contact with counselling service, excess of mortality and timing of the survey

b Model 2 (Relationship status and social life adjusted) is additionally adjusted for relationship status, trusted person availability, level of different social activities

c Model 3 (Socio-economic adjusted) = Model 1 + other variables (educational level of parents, tuition coverage)

d Model 4=Model 1+ all other models’ covariates

**Table S7**. Loss of economic resources and adjusted Prevalence Ratios for depressive symptoms (N=91,871), coding scheme 2, cut-off:9; COVID-19 International Student Well-being study, 23 countries worldwide, 2020

|  | **Model 1** ^a^ | **Model 2^b^** | **Model 3^c^** | **Model 4** ^d^ |
| --- | --- | --- | --- | --- |
|  | PR (95% CI) | PR (95% CI) | PR (95% CI) | PR (95% CI) |
| Equal | ref | ref | ref | ref |
| Decreased | 1.28 (1.23-1.34) | 1.26(1.21-1.31) | 1.28(1.23-1.33) | 1.26 (1.21-1.30) |

*Abbreviations: PR, Prevalence Ratio; 95% CI, 95% Confidence Interval.

a Model 1 is adjusted for age, sex, contact with counselling service, excess of mortality and timing of the survey

b Model 2 (Relationship status and social life adjusted) is additionally adjusted for relationship status, trusted person availability, level of different social activities

c Model 3 (Socio-economic adjusted) = Model 1 + other variables (educational level of parents, tuition coverage)

d Model 4=Model 1+ all other models’ covariates

**Table S8**. Loss of economic resources and adjusted Prevalence Ratios for depressive symptoms (N=91,871), coding scheme 3, cut-off:9; COVID-19 International Student Well-being study, 23 countries worldwide, 2020

|  | **Model 1** ^a^ | **Model 2^b^** | **Model 3^c^** | **Model 4** ^d^ |
| --- | --- | --- | --- | --- |
|  | PR (95% CI) | PR (95% CI) | PR (95% CI) | PR (95% CI) |
| Equal | ref | ref | ref | ref |
| Decreased | 1.24 (1.19-1.29) | 1.22(1.18-1.27) | 1.23(1.19-1.28) | 1.22 (1.18-1.26) |

*Abbreviations: PR, Prevalence Ratio; 95% CI, 95% Confidence Interval.

a Model 1 is adjusted for age, sex, contact with counselling service, excess of mortality and timing of the survey

b Model 2 (Relationship status and social life adjusted) is additionally adjusted for relationship status, trusted person availability, level of different social activities

c Model 3 (Socio-economic adjusted) = Model 1 + other variables (educational level of parents, tuition coverage)

d Model 4=Model 1+ all other models’ covariates

**Table S9.** Loss of economic resources and adjusted Prevalence Ratios for depressive symptoms (N=91,871), coding scheme 1, cut-off:3; COVID-19 International Student Well-being study, 23 countries worldwide, 2020

|  | **Model 1** ^a^ | **Model 2^b^** | **Model 3^c^** | **Model 4** ^d^ |
| --- | --- | --- | --- | --- |
|  | PR (95% CI) | PR (95% CI) | PR (95% CI) | PR (95% CI) |
| Coding scheme 1 | 1.03 (1.03-1.03) | 1.03 (1.03-1.03) | 1.03 (1.03-1.03) | 1.03 (1.03-1.03) |

*Abbreviations: PR, Prevalence Ratio; 95% CI, 95% Confidence Interval.

a Model 1 is adjusted for age, sex, contact with counselling service, excess of mortality and timing of the survey

b Model 2 (Relationship status and social life adjusted) is additionally adjusted for relationship status, trusted person availability, level of different social activities

c Model 3 (Socio-economic adjusted) = Model 1 + other variables (educational level of parents, tuition coverage)

d Model 4=Model 1+ all other models’ covariates

**Table S10**. Loss of economic resources and adjusted Prevalence Ratios for depressive symptoms (N=91,871), coding scheme 2, cut-off:3; COVID-19 International Student Well-being study, 23 countries worldwide, 2020

|  | **Model 1** ^a^ | **Model 2^b^** | **Model 3^c^** | **Model 4** ^d^ |
| --- | --- | --- | --- | --- |
|  | PR (95% CI) | PR (95% CI) | PR (95% CI) | PR (95% CI) |
| Equal | ref | ref | ref | ref |
| Decreased | 1.09 (1.09-1.10) | 1.09(1.08-1.09) | 1.09(1.08-1.10) | 1.09 (1.08-1.09) |

*Abbreviations: PR, Prevalence Ratio; 95% CI, 95% Confidence Interval.

a Model 1 is adjusted for age, sex, contact with counselling service, excess of mortality and timing of the survey

b Model 2 (Relationship status and social life adjusted) is additionally adjusted for relationship status, trusted person availability, level of different social activities

c Model 3 (Socio-economic adjusted) = Model 1 + other variables (educational level of parents, tuition coverage)

d Model 4=Model 1+ all other models’ covariates

**Table S11**. Loss of economic resources and adjusted Prevalence Ratios for depressive symptoms (N=91,871), coding scheme 3, cut-off:3; COVID-19 International Student Well-being study, 23 countries worldwide, 2020

|  | **Model 1** ^a^ | **Model 2^b^** | **Model 3^c^** | **Model 4** ^d^ |
| --- | --- | --- | --- | --- |
|  | PR (95% CI) | PR (95% CI) | PR (95% CI) | PR (95% CI) |
| Equal | ref | ref | ref | ref |
| Decreased | 1.08 (1.08-1.09) | 1.08(1.07-1.08) | 1.08(1.08-1.09) | 1.08(1.07-1.08) |

*Abbreviations: PR, Prevalence Ratio; 95% CI, 95% Confidence Interval.

a Model 1 is adjusted for age, sex, contact with counselling service, excess of mortality and timing of the survey

b Model 2 (Relationship status and social life adjusted) is additionally adjusted for relationship status, trusted person availability, level of different social activities

c Model 3 (Socio-economic adjusted) = Model 1 + other variables (educational level of parents, tuition coverage)

d Model 4=Model 1+ all other models’ covariates
